# Supplementary material for: The ubiquitin ligase Cul5 regulates CD4+ T cell fate choice and allergic inflammation
Source: Nat Commun. 2022 May 19;13:2786. doi: 10.1038/s41467-022-30437-x (PMC9120070; doi:10.1038/s41467-022-30437-x)
Supplement: Supplementary file 1 — Supplementary Information [file 41467_2022_30437_MOESM1_ESM.pdf]

Supplementary Information

Cul5 regulates $CD4^+$ T cell fate choice and allergic inflammation

Binod Kumar*[1,2], Natania S. Field*[1,3], Dale D. Kim[1], Asif A. Dar[1], Yanqun Chen[1,4], Aishwarya Suresh[1,5], Christopher F. Pastore[6], Li-Yin Hung[6], Nadia Porter[1], Keisuke Sawada[1,7], Palak Shah[1,8], Omar Elbulok[1,9], Emily K. Moser[1,10], De'Broski R. Herbert[6] and Paula M. Oliver[1,11,12]

* Contributed equally

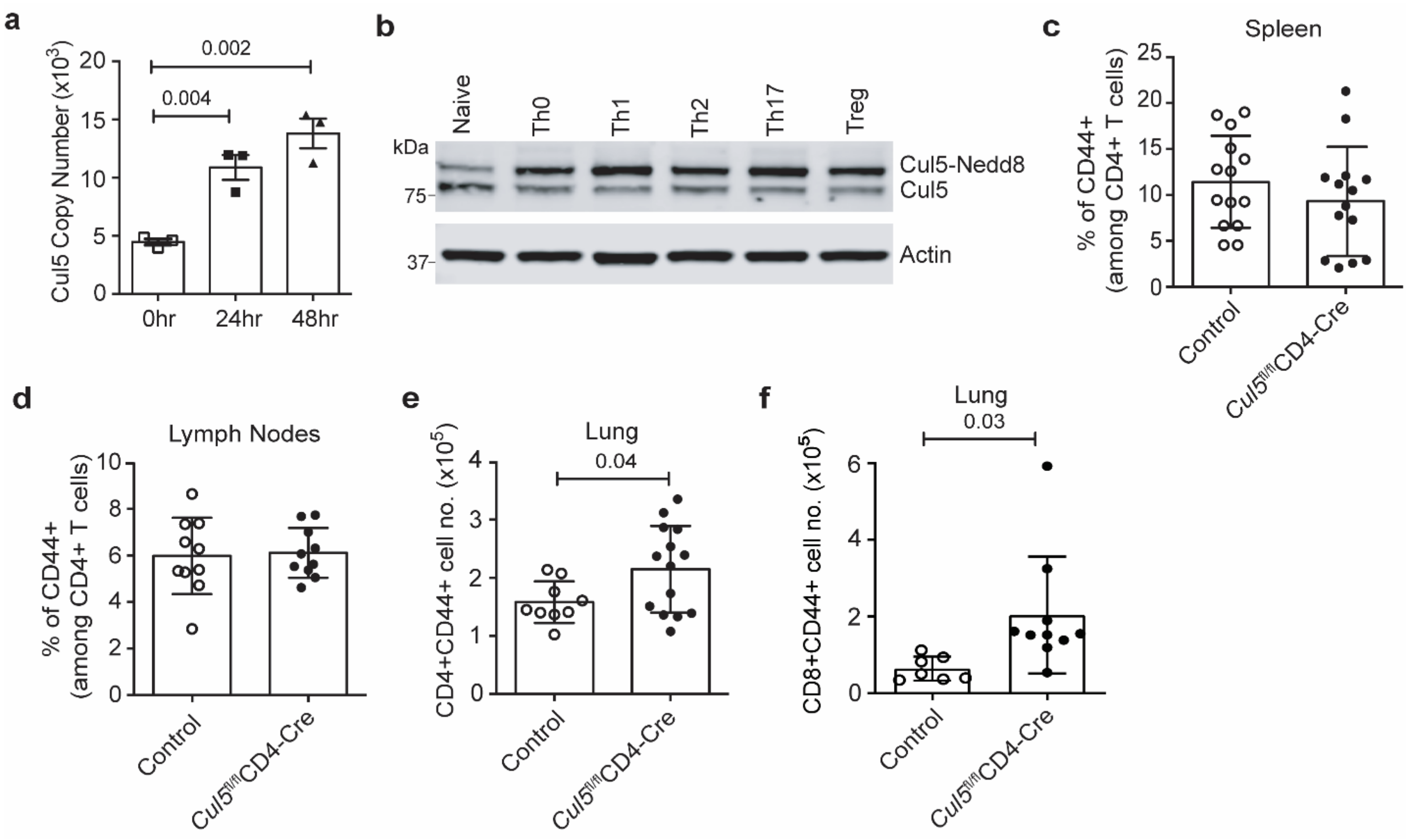

Supplementary Fig. 1: Cul5 expression and neddylation is increased in CD4$^+$ T cells following stimulation

8-10-week old biologically independent animal were used for experiments. **a** Cul5 copy number in CD4$^+$ T cells. CD4$^+$ T cells were stimulated for 0, 24, and 48hrs by anti-CD3 and anti-CD28[1]. n=3 biologically independent cell examined over one independent experiment. Copy number was calculated using proteomic ruler[2]. **b** Immunoblot analysis of Cul5 in murine naïve CD4$^+$ T cells cultured under different polarizing condition. On day 5 cells were restimulated with anti-CD3 and anti-CD28 for 4hrs, lysed and probed with indicated proteins. n=2, examined over two independent experiments. **c** Frequency of CD4$^+$CD44$^+$ T cells in spleen of control and *Cul5*$^{fl/fl}$CD4-Cre mice. n=14, examined over

seven independent experiments. **d** same as (d) for lymph nodes. n=10, examined over five independent experiments. **e** same as (c) for lungs. n=9 (control); 14 (*Cul5*$^{fl/fl}$CD4-Cre) examined over five independent experiments. **f** CD8^{+}CD44^{+} T cell numbers in lungs. n=7(control); 10(*Cul5*$^{fl/fl}$CD4-Cre) examined over three independent experiments. Data is presented as Mean$\pm$SD in panel **a**, and **c- f**. *P* value was calculated by unpaired two tailed *t* test.

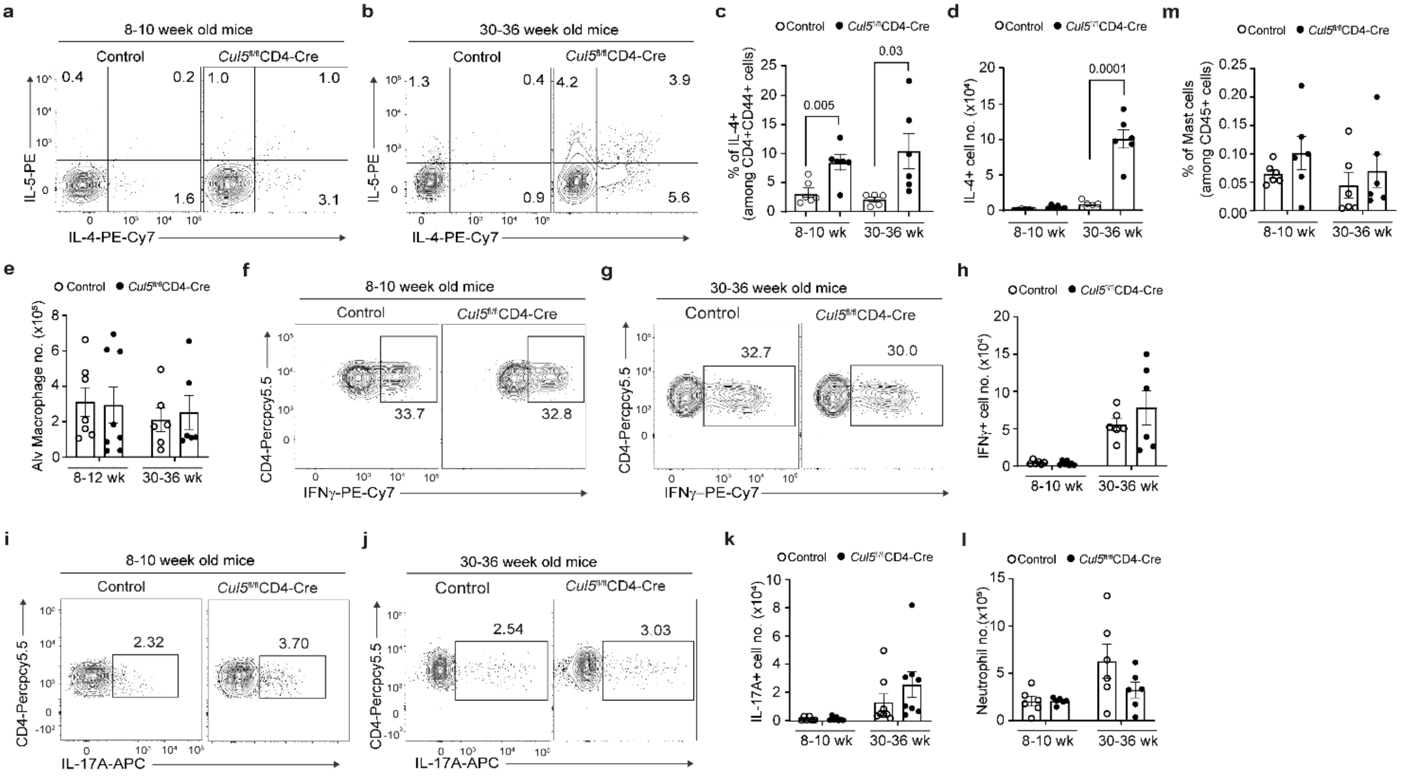

Supplementary Fig. 2: *Cul5*$^{fl/fl}$CD4-Cre mice had comparable level of Th1 and Th17 cells in lung

Biologically independent 8-10 and 30-36 week old control and *Cul5*$^{fl/fl}$CD4-Cre mice were analyzed. **a-b** Representative flow plots showing IL-4^{+} and IL-5^{+} in lungs. Compiled data shown in panel **(c)** **c** Compiled data showing the frequency of IL-4^{+} cells. For 8-10-week old n=6; for 30-36 week old n=5 (control); 6(*Cul5*$^{fl/fl}$CD4-Cre) examined over two independent experiments). **d** Compiled data showing the number of IL-4^{+} cells. n=5 (control); 6(*Cul5*$^{fl/fl}$CD4-Cre) examined over two independent experiments). **e** Numbers of alveolar macrophages in lungs. For 8-10-week old n=7 (control); 6(*Cul5*$^{fl/fl}$CD4-Cre); for 30-36 week old n=6 examined over two independent experiments. **f-g** Representative

flow plots showing IFNγ^+ cells in lungs. Compiled data shown in panel **(h)**. **h** Compiled data showing the number of IFNγ^+ cells. For 8-10-week old n=6 (control); 7(*Cul5*$^{fl/fl}$CD4-Cre); for 30-36 week old n=6 examined over two independent experiments. **i-j** Representative flow plots showing IL-17A$^+$ cells in lungs. **k** Compiled data showing the number of IL-17A$^+$ cells. For 8-10-week old n=6 (control); 7(*Cul5*$^{fl/fl}$CD4-Cre); for 30-36 week old n=8 examined over two independent experiments. **l** Frequencies of neutrophils in the lungs. n=6 examined over two independent experiments. **m** Frequencies of mast cells in lungs. n=6 examined over two independent experiments. Data is presented as Mean±SEM in panel **c-e**, **h, k-m**. *P* value was calculated using unpaired two tailed *t* test.

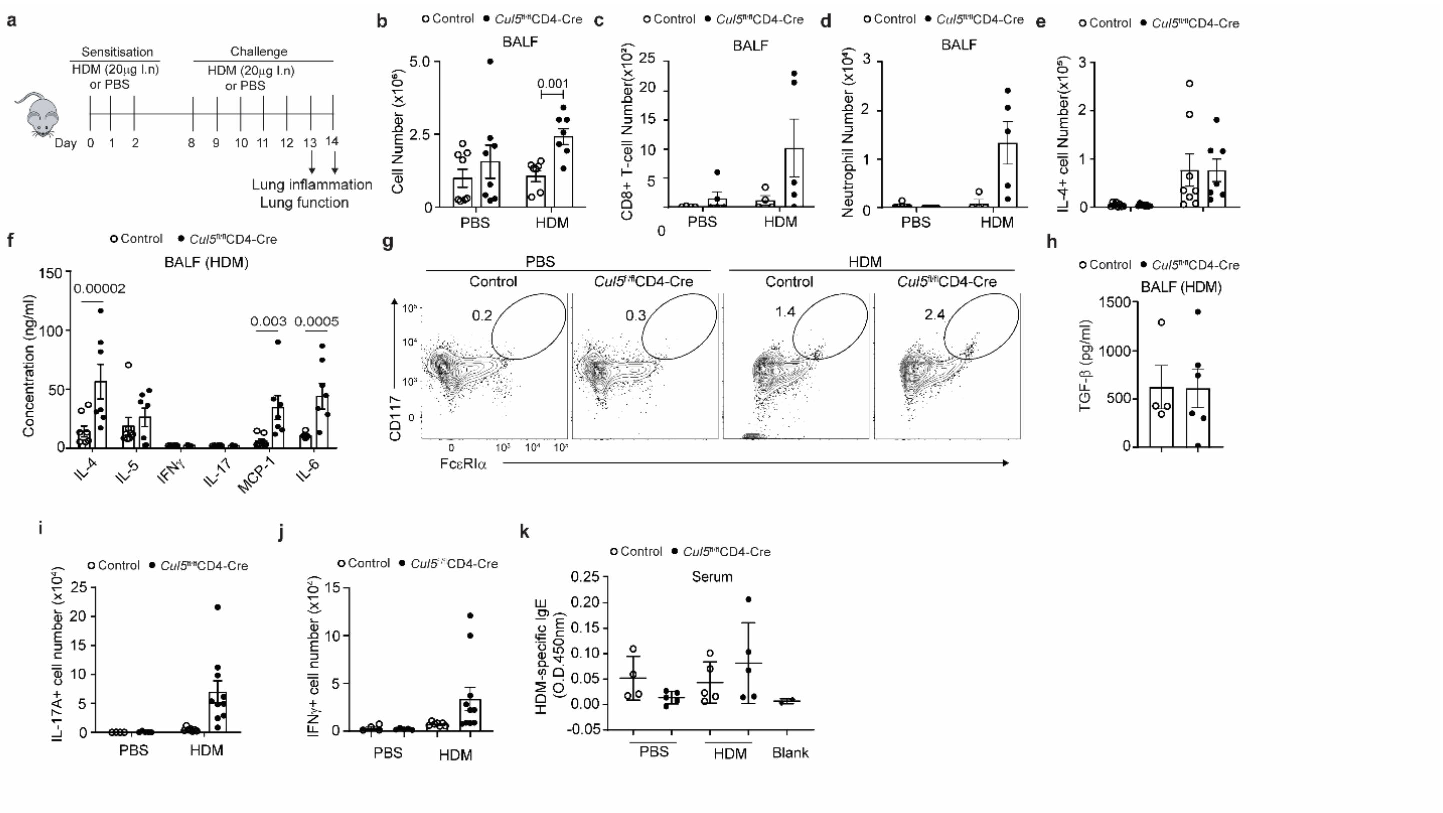

Supplementary Fig.3: *Cul5*$^{fl/fl}$CD4-Cre mice show increased Th2- and -Th9 inflammation upon HDM challenge

Biologically independent 8-10-week old control and *Cul5*$^{fl/fl}$CD4-Cre mice were treated with either PBS or HDM. **a** House Dust Mite (HDM) exposure protocol. **b** Total number of cells present in Broncho Alveolar Lavage Fluid (BALF). For PBS group, n=8; for HDM group, n=7 examined in two independent experiments. **c-d** Total numbers of $CD8^+$ T cells **(c)** and neutrophils **(d)** present in BAL. For PBS group, n=4; for HDM group, n=5 examined in one independent experiment. **e** Assessment of cytokines in BAL fluid by ELISA. For PBS group, n=8; for HDM group, n=7 examined in two independent experiments. **f** Number of IL-4^+ cells in the lungs. For PBS group, n=8; for HDM group,

n=7 examined over two independent experiments. **g** Representative flow plots showing the frequency of mast cells in lungs. For PBS group, n=4 (control); 5 (*Cul5*$^{fl/fl}$CD4-Cre); for HDM group n=7 (control); 10 (*Cul5*$^{fl/fl}$CD4-Cre) examined over two independent experiments. **h** TGF-β levels in the BALF of mice. n=4 (control); 6 *Cul5*$^{fl/fl}$CD4-Cre) examined in one independent experiment. **j** Consolidated data showing the number of IL-17A^{+} cells in lungs. For PBS group, n=4 (control), 5 (*Cul5*$^{fl/fl}$CD4-Cre); for HDM group, n=7 (control); 10 (*Cul5*$^{fl/fl}$CD4-Cre) examined in two independent experiments. **k** Consolidated data showing the number of IFNγ^{+} cells from the lungs. For PBS group, n=4 (control), 5 (*Cul5*$^{fl/fl}$CD4-Cre) examined in one independent experiment. For HDM group, n=7 (control); 10 (*Cul5*$^{fl/fl}$CD4-Cre) examined over two independent experiments. **l** HDM specific IgE levels in the serum. For PBS group, n=4 (control), 5 (*Cul5*$^{fl/fl}$CD4-Cre); for HDM group n=5 examined over one independent experiments. Data is presented as Mean±SEM in panel **b-f**, **h-k**. *P* value was calculated using unpaired two tailed *t* test.

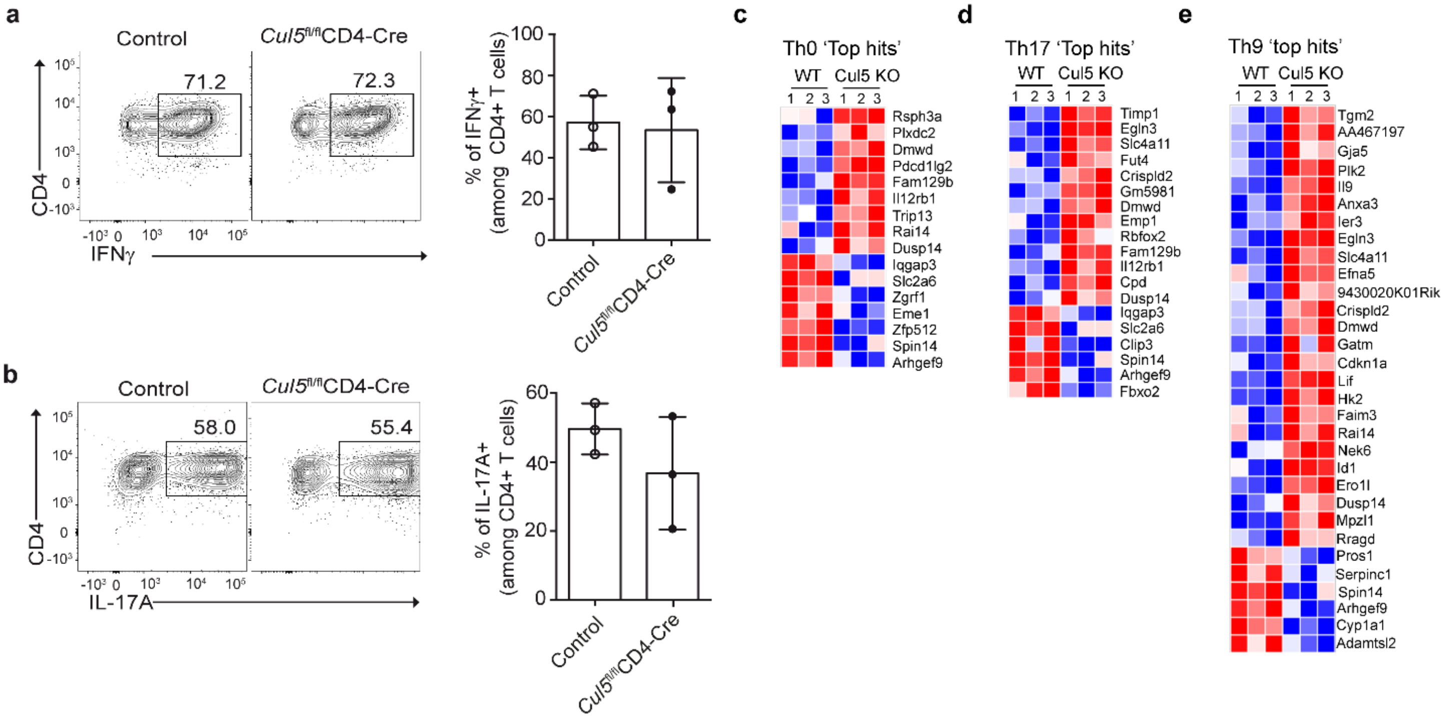

Supplementary Fig. 4: Th1 or Th17 differentiation appears to be independent of Cul5

Naïve CD4$^+$ T cells from biologically independent 8-10-week old control and *Cul5*$^{fl/fl}$CD4-Cre mice were used for experiments. **a-b** Representative flow plots and consolidated data (on right) of WT and *Cul5*$^{fl/fl}$CD4-Cre cells showing the frequency of Th1 (a) and Th17 (b) cells.n=3 examined over three independent experiments. **c-d** Heatmap of genes classified as top hit genes in Th17 (c) and Th0 (d) subtypes and identified in WT and *Cul5*$^{fl/fl}$CD4-Cre CD4$^+$ T cells (n=3 examined in one independent experiment). **e** Heatmap of genes classified as top hit Th9 genes using microarray data (GSE44937) and identified in WT and *Cul5*$^{fl/fl}$CD4-Cre CD4$^+$ T cells. Data is presented as Mean+SD in panels **a-b.**

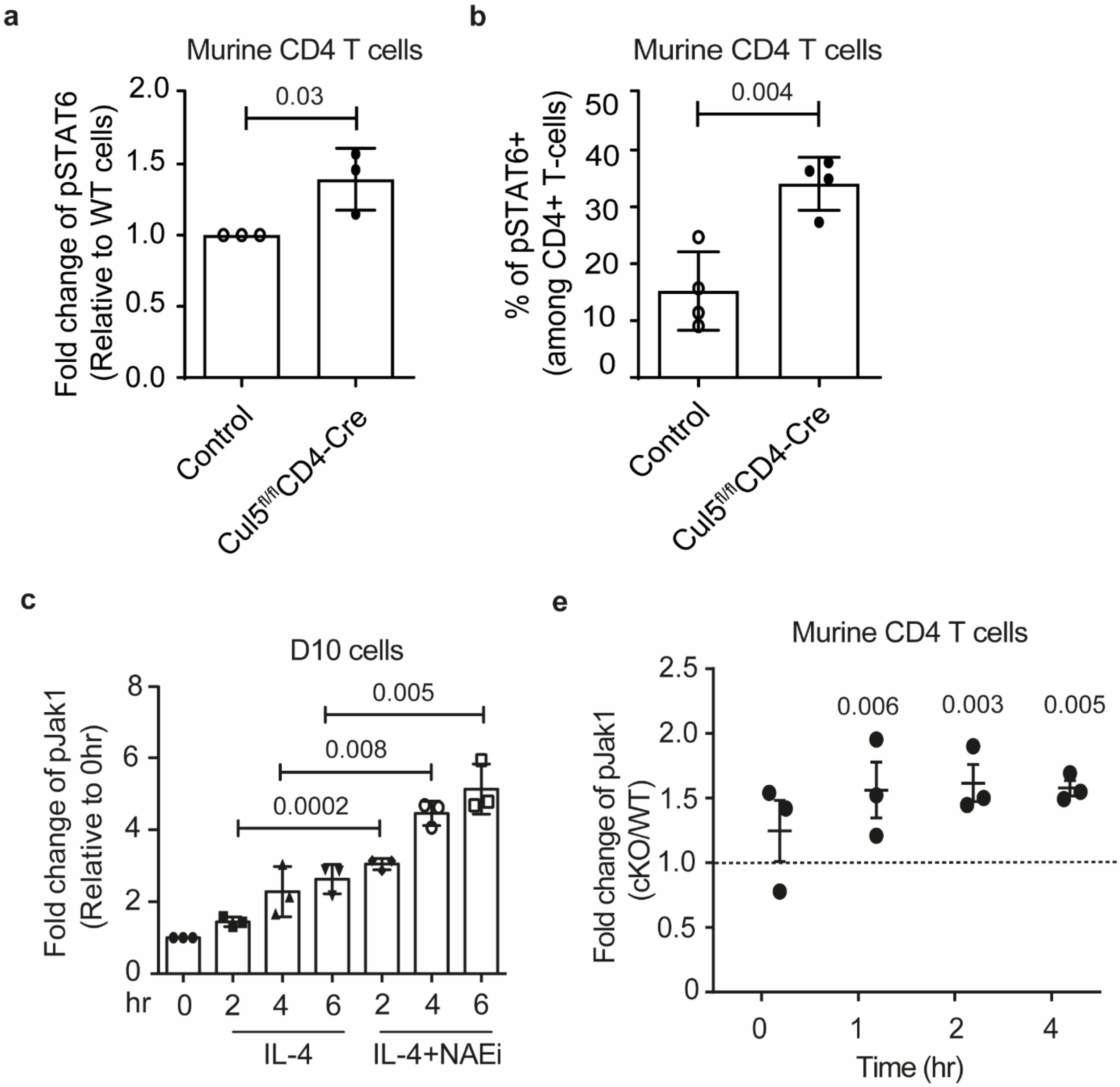

Supplementary Fig. 5. Cul5 acts as a rheostat for pJak1 activation in CD4⁺ T cells

a Quantification of immunoblot in 6a. Graph represents the fold change of pSTAT6 in Cul5 deficient CD4⁺ T cells compared to control. Data was quantitated using Image Studio

Software. The fold change was calculated by adjusting pSTAT6 level in control to 1 and then relative change in pSTAT6 level in Cul5 KO was calculated accordingly. n=3 biologically independent cells examined over two independent experiments**. b** Compiled data showing the percentage of $CD4^+$ T cells that are $pSTAT6^+$ upon IL-4 stimulation as assessed by flow cytometry. n=4 biologically independent cells examined over two independent experiments. **c** Fold change of pJak1 in D10 cells treated with IL-4, or IL-4 in combination with NAEi for indicated times. To calculate fold changes pJak1 at 0hr was set as 1 and relative pJak1 was calculated for each time point. n=3 biologically independent cells examined over three independent experiments. **d** Fold change of pJak1 in Cul5-deficient $CD4^+$ T cells compared to control cells upon IL-4 treatment. Levels of pJak1 for control was set as 1 for each time point and relative fold change in Cul5 deficient $CD4^+$ T cells was calculated. n=3 biologically independent cells examined over three independent experiments. Data is presented as Mean ± SEM in panel **a-e**. *P* value was calculated using unpaired two tailed *t*-test.

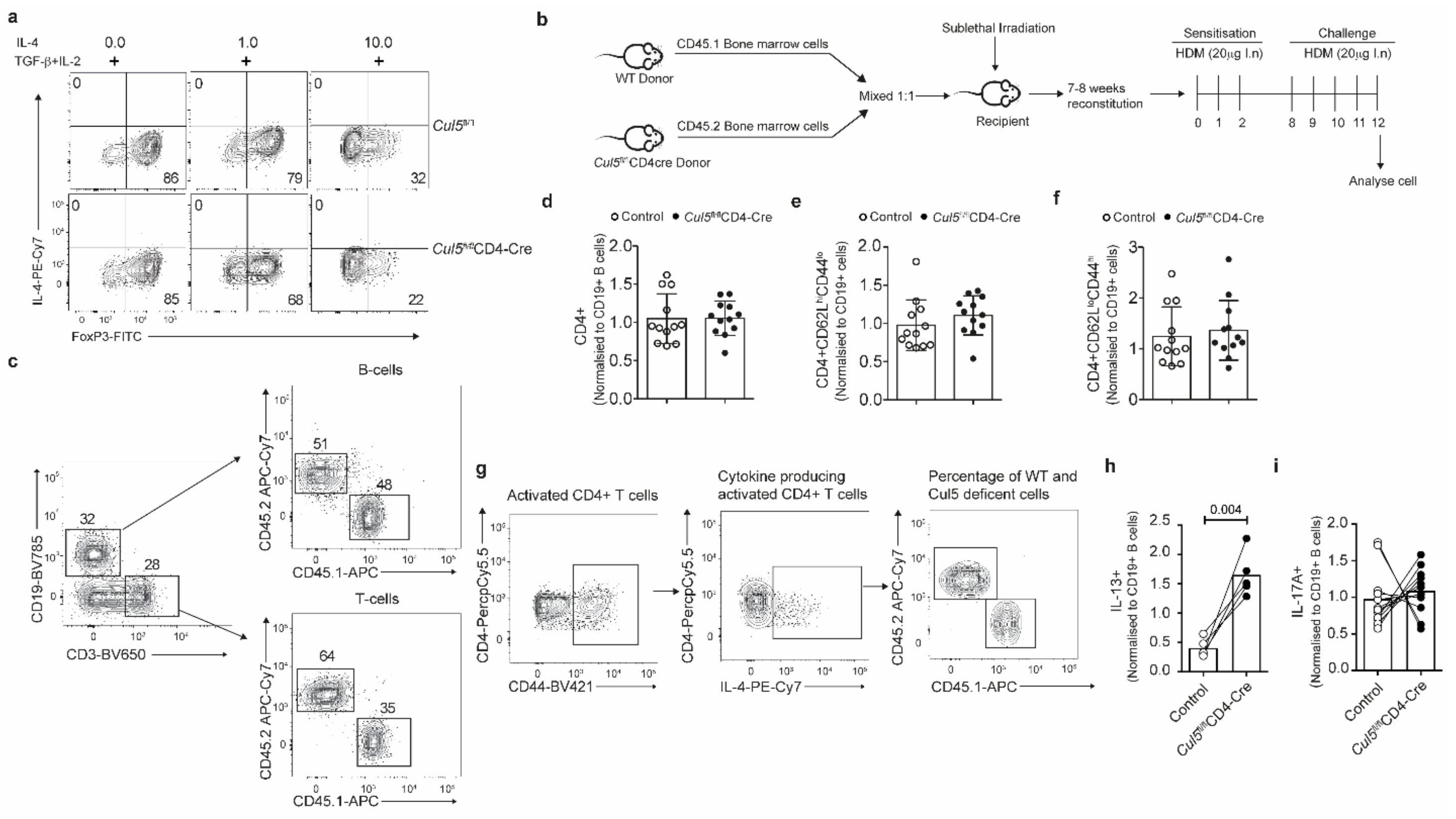

Supplementary Fig. 6: WT and Cul5 deficient CD4$^+$ T cells are similarly reconstituted in mixed bone marrow chimera mice

a Representative flow plots showing the frequencies of IL-4$^+$ and Foxp3$^+$ cells from WT and Cul5 deficient CD4$^+$ T cells. n=3 examined over two independent experiments. **b** Schematics of the mixed bone marrow chimera experiment. **c** Representative flow plot showing the gating strategy used to determine the reconstitution of B and T cells. **d** Bar graph shows the relative ratios of CD4$^+$ T cells from CD45.1$^+$ (WT) and CD45.2$^+$ (Cul5 KO) donors. Ratio was calculated by dividing the percentages of CD4$^+$ T cells of each

genotype with the percentages of B cells from the same genotype to account for reconstitution. n=12 biologically independent recipient mice examined over two independent experiments. Two pair of donors were used. **e-f** shows the relative proportion of naïve ($CD4^{+}CD62L^{hi}CD44^{lo}$) (**e**) and effector ($CD4^{+}CD62L^{lo}CD44^{hi}$) (**f**) $CD4^{+}$ T cells that are CD45.1 (WT) or CD45.2 (Cul5 deficient). n=12 biologically independent recipient mice examined over 2 independent experiments. Two pair of donors were used. **g** Flow cytometry gating strategy used to determine the relative proportion of cytokine producing $CD4^{+}CD44^{+}$ T cells. **h** Compiled data showing the proportion of IL-13^{+} cells from CD45.1 (WT donors) or CD45.2(Cul5 deficient donors). n=5 biologically independent recipient mice examine in one independent experiment. One pair of donor was used. **i** Compiled data showing the proportion of IL-$17A^{+}$ cells from CD45.1 (WT donors) or CD45.2(Cul5 deficient donors). n=12 biologically independent recipient mice were examined over 2 independent experiments. Two pair of donors were used.. To calculate the relative ratios of donor IL-13^{+} or IL-$17A^{+}$ CD45.1 or CD45.2 was divided by CD45.1 or CD45.2 $CD19^{+}$ cells to account for reconstitution. Data is presented as Mean+SEM in panel **d-f.** In panel **h-i** data is presented as mean. *P* value was calculated by pair two tailed *t* test.

Supplementary Fig 7: Gating strategy to identify CD4 and CD8 T cells in thymus (used in Figure 1e and f).

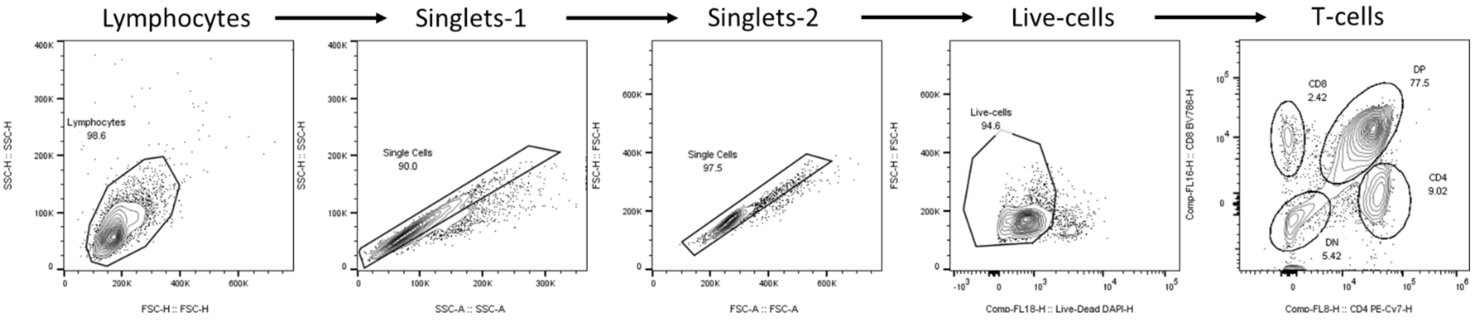

Supplementary Fig 8: Gating strategy to identify activated CD4⁺ T cells (used in Figure 1g, h and supplementary Figure 1).

.

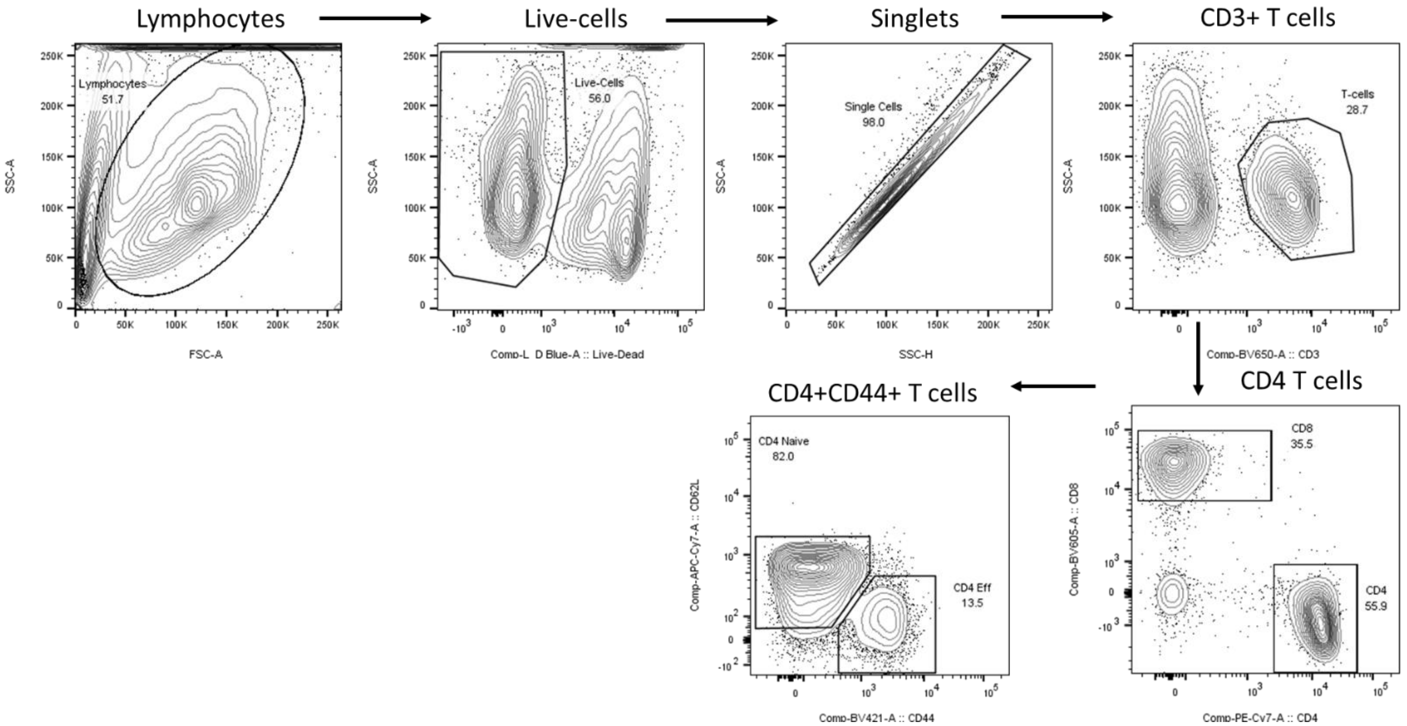

Supplementary Fig 9: Gating strategy to identify cytokine producing CD4+ T cells (used in Figure 2e-j, 3i-n, 7f-h, Supplementary Figure 2a-k, Supplementary figure 3e,i, j, Supplementary figure 6h and i).

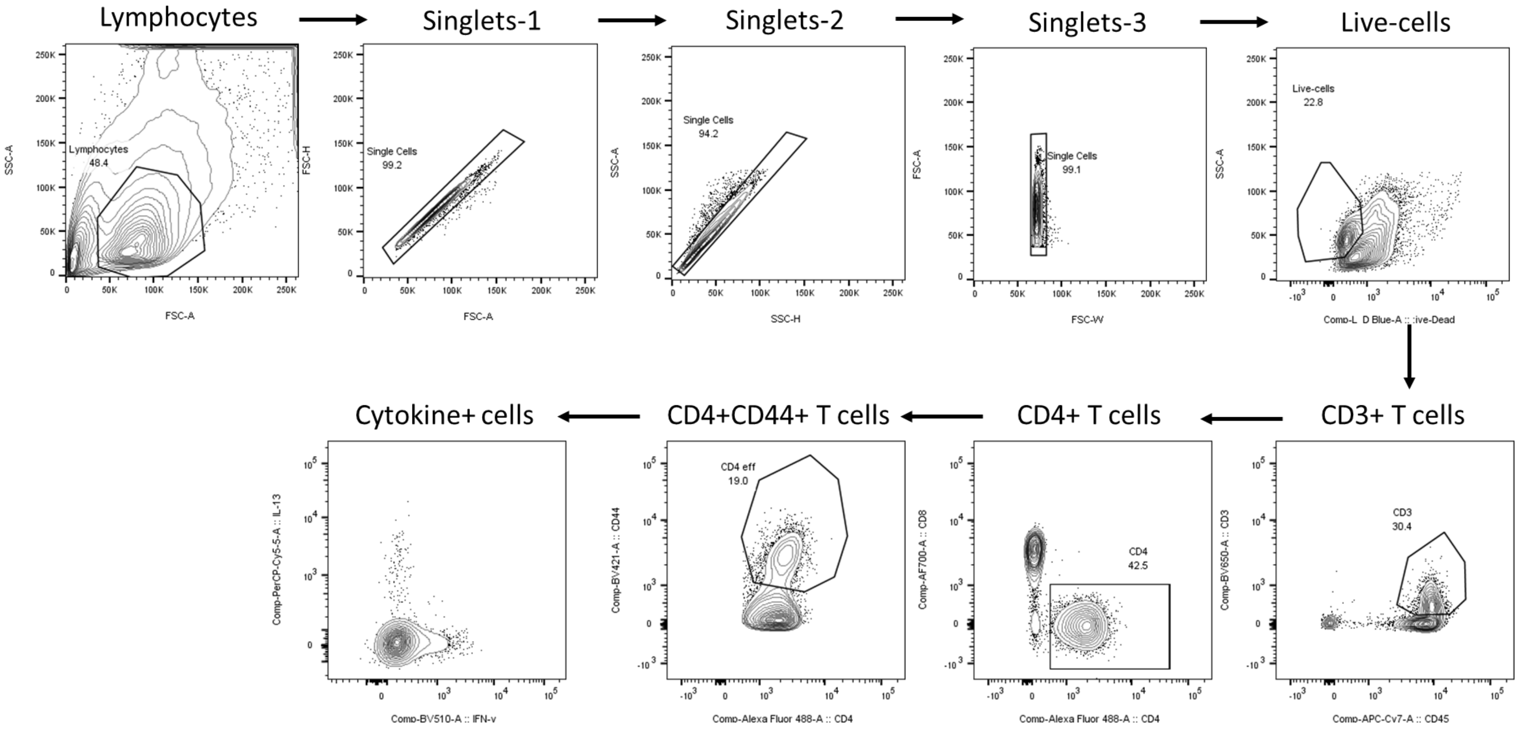

Supplementary Fig 10: Gating strategy to identify mast cells (used in Figure 3o, p, Supplementary Figure 2m and Supplementary Figure 3g)

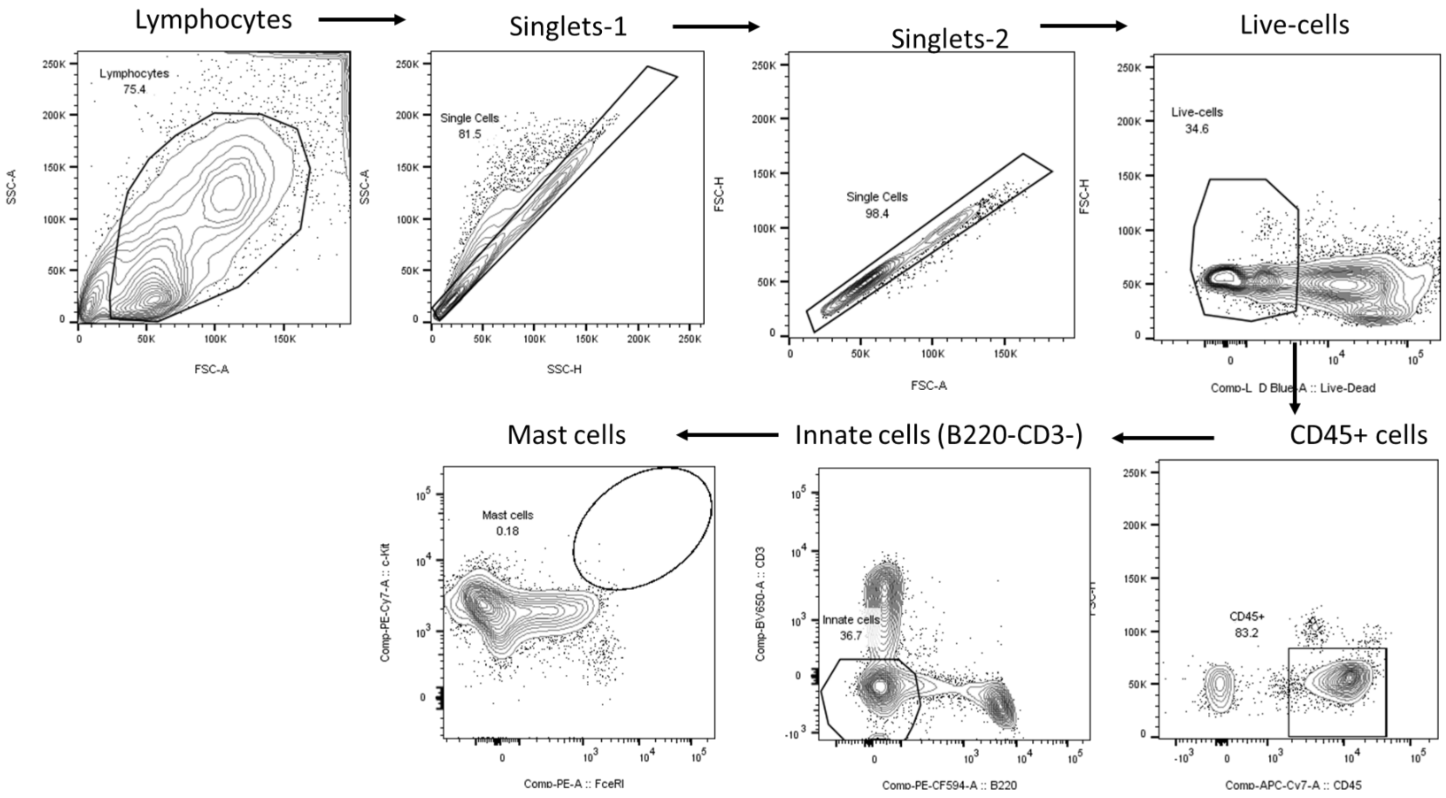

Supplementary Fig 11: Gating strategy to identify Eosinophils, Alveolar Macrophages and Neutrophils (used in Figure 2 and 3)

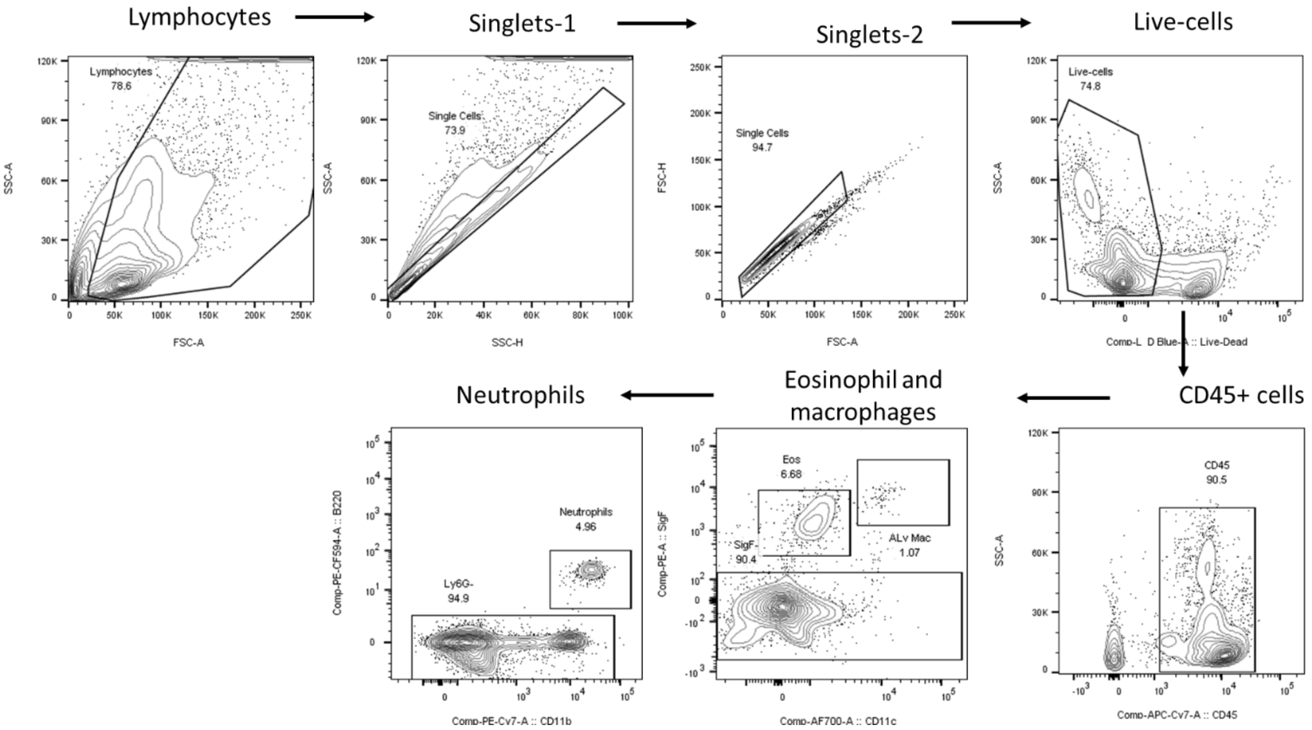

Supplementary Fig 12: Gating strategy to identify pSTAT6 level (used in Figure 6)

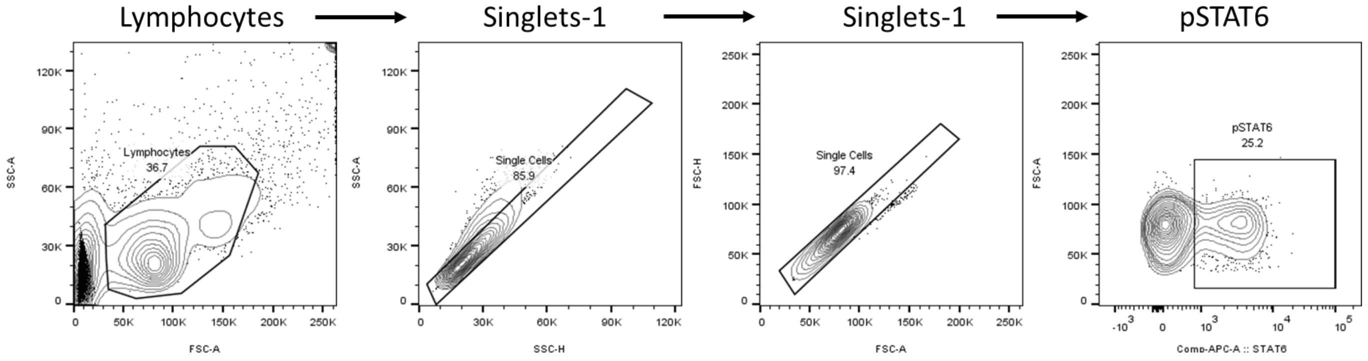

References:

1. Field, N.S. *et al.* Itch attenuates CD4 T-cell proliferation in mice by limiting WBP2 protein stability. *Eur J Immunol* **50**, 1468-1483 (2020).

2. Wisniewski, J.R., Hein, M.Y., Cox, J. & Mann, M. A "proteomic ruler" for protein copy number and concentration estimation without spike-in standards. *Mol Cell Proteomics* **13**, 3497-3506 (2014).
