# Supplementary material for: The ubiquitin ligase Cul5 regulates CD4+ T cell fate choice and allergic inflammation
Source: Nat Commun. 2022 May 19;13:2786. doi: 10.1038/s41467-022-30437-x (PMC9120070; doi:10.1038/s41467-022-30437-x)
Supplement: Supplementary file 3 — Reporting Summary [file 41467_2022_30437_MOESM3_ESM.pdf]

nature research

Corresponding author(s): Paula M Oliver

Last updated by author(s): Mar 29, 2022

Reporting Summary

Nature Research wishes to improve the reproducibility of the work that we publish. This form provides structure for consistency and transparency in reporting. For further information on Nature Research policies, see our Editorial Policies and the Editorial Policy Checklist.

Statistics

For all statistical analyses, confirm that the following items are present in the figure legend, table legend, main text, or Methods section.

n/a	Confirmed	
☐	☒	The exact sample size (*n*) for each experimental group/condition, given as a discrete number and unit of measurement
☐	☒	A statement on whether measurements were taken from distinct samples or whether the same sample was measured repeatedly
☐	☒	The statistical test(s) used AND whether they are one- or two-sided<br>*Only common tests should be described solely by name; describe more complex techniques in the Methods section.*
☐	☐	A description of all covariates tested
☐	☒	A description of any assumptions or corrections, such as tests of normality and adjustment for multiple comparisons
☐	☒	A full description of the statistical parameters including central tendency (e.g. means) or other basic estimates (e.g. regression coefficient) AND variation (e.g. standard deviation) or associated estimates of uncertainty (e.g. confidence intervals)
☐	☒	For null hypothesis testing, the test statistic (e.g. *F*, *t*, *r*) with confidence intervals, effect sizes, degrees of freedom and *P* value noted<br>*Give P values as exact values whenever suitable.*
☒	☐	For Bayesian analysis, information on the choice of priors and Markov chain Monte Carlo settings
☒	☐	For hierarchical and complex designs, identification of the appropriate level for tests and full reporting of outcomes
☒	☐	Estimates of effect sizes (e.g. Cohen's *d*, Pearson's *r*), indicating how they were calculated

Our web collection on statistics for biologists contains articles on many of the points above.

Software and code

Policy information about availability of computer code

Data collection	BD LSR Fortessa with FACSDiva software<br>Li-COR with ImageStudio software
Data analysis	FlowJo Version 10<br>GraphPad Prism 7 and 9<br>Imagestudio Lite<br>Microsoft Excel<br>Kallisto<br>R studio<br>Morpheus

For manuscripts utilizing custom algorithms or software that are central to the research but not yet described in published literature, software must be made available to editors and reviewers. We strongly encourage code deposition in a community repository (e.g. GitHub). See the Nature Research guidelines for submitting code & software for further information.

Data

Policy information about availability of data

All manuscripts must include a data availability statement. This statement should provide the following information, where applicable:

- Accession codes, unique identifiers, or web links for publicly available datasets
- A list of figures that have associated raw data
- A description of any restrictions on data availability

The RNAseq raw data are deposited and available from the GEO database under the accession number GSE184321. IP-MS/MS data from D10 cells is deposited and

available from PRIDE database under accession number PXD028492.

Field-specific reporting

Please select the one below that is the best fit for your research. If you are not sure, read the appropriate sections before making your selection.

☒ Life sciences ☐ Behavioural & social sciences ☐ Ecological, evolutionary & environmental sciences

For a reference copy of the document with all sections, see nature.com/documents/nr-reporting-summary-flat.pdf

Life sciences study design

All studies must disclose on these points even when the disclosure is negative.

Sample size	No sample size calculation was performed. Sample size was based on the experimental aim with genotype difference in most experiments. The size of the group and number of replicates depended on the magnitude of the effect being observed and sample to sample variation in the parameters being measured. Sample size were deemed sufficient based on the reproducibility between independent experiments. Mice of both sexes were used for experiments. Detailed number for individual key experiments is shown below.<br><br>Immunophenotyping: Sample size was determine based on prior experience and published literature (Aki et al. Nat Imm 2018). Based on our prior experience and published literature suggesting that frequency of Th2 cells are very low at steady state we estimated that samples size of 3-20 should be sufficient to reach conclusions.<br><br>HDM experiments: Based on published literature (woo et al. Sci Rep 2018), we selected 3-4 mice in each group of individual genotype and repeated experiment two times. 3-4 mice mice in each group was included to reach a statistical conclusion.<br><br>RNAseq experiment: Three mice from each genotype was used to reach a statistical conclusions.<br><br>IP-MS/MS experiment: Based on prior experience (Dar et al. 2021 Plos Biol) we repeated this experiment two times. To assure reproducibility we performed Immunoprecipitation in the same condition with anti-Cul5 antibody followed by western blotting for CIS or Jak1 to confirm that these proteins interact with each other as suggested by IP-MS/MS data.<br><br>TUBE Pulldown: Experiment was repeated two times under same condition to check ubiquitination of pJak1. We based our number of replicate on the basis degree of difference observed. No statistical method was used to calculate the number of replicates.<br><br>Western Blotting: Each experiment was repeated at least three times to assure reproducibility. We based our number of replicate on the basis degree of difference observed and statistics. No statistical method was used to calculate the number of replicates.
Data exclusions	Flow data that showed staining issue and Immunoblotting data that showed either unequal loading or bands for protein of interest were not clearly distinguishable from background were excluded.
Replication	Each experiment was repeated at least two times to assure reproducibility. All attempts for replication was successful. Mast cell data for PBS treated group is from one experiment with 4-5 mice per group. The rationale for one replicate is based on the result that in PBS treated group there was no significant difference between two genotype even though we had more than three animals per genotype.
Randomization	Litter mate controls were used in most experiments and randomly assigned to groups. In certain experiments, CD45.1 or CD45.1/CD45.2 wild type mice were used. In all experiment animals were gender matched and age matched.
Blinding	For measuring airway resistance researchers were blinded. For rest of experiments described in the manuscript investigators were not blinded in this study (as is accepted in the field) as blinding is not needed for a scientifically sound result. For mouse experiments it is required to provide a cage label and mice number for each experimental cage detailing the conditions each individual mouse has received, and each mouse needs to be identifiable with a ear marking, which prevents blinding in such experiments.

Reporting for specific materials, systems and methods

We require information from authors about some types of materials, experimental systems and methods used in many studies. Here, indicate whether each material, system or method listed is relevant to your study. If you are not sure if a list item applies to your research, read the appropriate section before selecting a response.

Materials & experimental systems

n/a	Involved in the study
	☒ Antibodies
	☒ Eukaryotic cell lines
☒	Palaeontology and archaeology
	☒ Animals and other organisms
☒	Human research participants
☒	Clinical data
☒	Dual use research of concern

Methods

n/a	Involved in the study
☒	ChIP-seq
	☒ Flow cytometry
☒	MRI-based neuroimaging

Antibodies

Antibodies used

Flow antibodies/dilutions
anti-CD4 (BioLegend, clone GK1.5, cat no. 100412, 1/200).
anti-CD19 (Biolegend, 1D3/CD19, cat no. 152410, 1/200).
anti-CD3 (BioLegend or BD clone 17A2, cat no.100236/555273, 1/200).
anti-CD62L (BioLegend, clone MEL-14, cat no.104412, 1/200).
anti-CD44 (BioLegend, clone IM7, cat no. 103012, 1/200).
anti-CD45.1 (BioLegend, clone A20, cat no.110714, 1/400).
anti-CD45.2 (BioLegend, clone 104, cat no. 109814, 1/400).
anti-CD45 (Biolegend, clone 30 F-11, cat no. 103112, 1/400).
anti-FceRI (ebiosciences, clone Mar-1, cat no. 14-5898-82, 1/100).
anti-CD117 (Biolegend, clone 2-B8, cat no. 313206, 1/100).
anti-Siglec F (BD, clone E50-2440, cat no.552126, 1/200).
anti-CD11c (Biolegend, clone N418, cat no. 117310, 1/200).
anti-CD11b (Biolegend, clone M1/70 cat no. 101212, 1/200).
anti-IFN-y (BD, clone XMG1.2 cat no. 505850, 1/100).
anti-IL2 (BL, clone JES6-5H4 cat no. 503809, 1/100).
anti-IL5 (BD, clone TRFK5 cat no. 554396, 1/100).
anti-IL4 (BD, clone 11B11 cat no. 562044, 1/100).
anti-IL13 (ebioscience; clone eBio13A cat no. 53-7133-82, 1/100).
anti-IL17A (BL, clone TC11-18H10.1 cat no. 5069161, 1/100).
antiIL-9 (BL, clone RM9A4 cat no. 507604, 1/100).

Western Blotting antibodies/dilutions
anti-Cul5 (Bethyl laboratories, Cat No. A302-173A, 1/5000).
CIS (CST, Cat No. 8731S and SCBT Cat No. 166326, 1/1000 for CST and 1/100 for SCBT).
pJak1 (CST, Cat No. 74129 and 3331, 1/1000).
Jak1 (BD, Cat No. 610231BD clone 73, 1/1000).
pSTAT6 (CST, Cat No. 56554, 1/5000).
STAT6 (CST, Cat No. 5397, 1/5000).
antiIL4Ra (BD, Cat No. PA5-38614, 1/500)
beta-Actin (SCBT, Cat No. SC4778, 1/10000).

Validation

Antibody validation statement by vendor(s) are described below:.

Flow antibodies:
anti-CD4. C57BL/6 mouse splenocytes were stained with CD4 (clone GK1.5)
anti-CD19. C57BL/6 mouse splenocytes were stained with CD19 APC (clone 1D3/CD19).
anti-CD3. C57BL/6 mouse splenocytes were stained with CD3 (clone 17A2)(Biolegend); This antibody is routinely tested by flow cytometric analysis (BD).
anti-CD62L. C57BL/6 mouse splenocytes were stained with CD62L (clone MEL-14)
anti-CD44. C57BL/6 mouse splenocytes stained with IM7 APC
anti-CD45.1. SJL mouse splenocytes stained with A20 APC
anti-CD45.2. C57BL/6 mouse splenocytes stained with 104 APC
anti-CD45. C57BL/6 mouse splenocytes stained with 30-F11 APC
anti-FceRI. Surface staining of MC/9 cell line with Anti-Mouse Fc epsilon Receptor I alpha (Fc epsilon R1) FITC (left) and PE (right).Appropriate isotype controls were used
anti-CD117. Human erythroleukemia cell line TF-1 stained with 104D2 APC
anti-Siglec F. The E50-2440 monoclonal antibody specifically recognizes Siglec-F
anti-CD11c. C57BL/6 mouse splenocytes stained with N418 APC and PK136 PE
anti-CD11b. C57BL/6 mouse bone marrow cells were stained with CD11b (clone M1/70) APC
anti-IFNy. C57BL/6 mouse splenocytes were stimulated with Cell Activation Cocktail (with Brefeldin A) for 6 hours, stained with CD3 Alexa Fluor® 488, fixed with Fixation Buffer, permeabilized with Intracellular Staining Permeabilization Wash Buffer (10X), and then

intracellularly stained with IFN-γ (clone XMG1.2) APC/Cyanine7
anti-IL2. PMA+ionomycin stimulated C57BL/6 mouse splenocytes (6 hours) stained with anti-CD3 PE (17A2) and intracellularly stained with JES6-5H4 APC
anti-IL5. The TRFK5 antibody reacts with mouse interleukin-5 (IL-5) and cross-reacts with human IL-5
anti-IL4. Expression of IL-4 by stimulated CD4+ and CD4-BALB/c spleen cells.
anti-IL13. Intracellular staining of Th2-polarized and restimulated CD4+ splenocytes with Anti-Mouse CD4 PerCP-eFluor® 710 (Product # 46-0042-82) and 0.125 μg of Rat IgG1 K Isotype Control Alexa Fluor® 488 (Product # 53-4301-80) (left) or 0.125 μg of Anti-Mouse IL-13 Alexa Fluor® 488
anti-IL17A. PMA (20 ng/ml) + ionomycin (1 μg/ml) -stimulated (6 hours + monensin, 2 μM) mouse thymoma cell line EL-4 intracellularly stained with TC11-18H10.1 APC
antiIL-9. Enriched human CD4+ T cells were stimulated with PMA+ionomycin, then intracellular stained with anti-IFN-γ (4S.B3) FITC and MH9A4 PE

Western Blotting antibodies:
anti-Cul5. Whole cell lysate (10 μg) from NIH 3T3, CT26, and CH27 cells prepared using NETN lysis buffer. Affinity purified rabbit anti-Cul5 antibody A302-173A (lot A302-173A-2) used for WB at 0.1 μg/ml.

CIS. Western blot analysis of extracts from NK-92 cells, untreated (-) or treated (+) with Human Interleukin-2 (hIL-2) #8907 (10 ng/ml, overnight), or BaF3 cells, untreated (-) or treated (+) with Mouse Interleukin-3 (mIL-3) #8923 (10 ng/ml, 6 hr), using CISH (D4D9) Rabbit mAb (upper) and β-Actin (D6A8) Rabbit mAb #8457 (lower) (CST). Western blot analysis of CIS expression in non-transfected: sc-117752 (A) and mouse CIS transfected: sc-119271 (B) 293T whole cell lysates (SCBT).

pJak1. Western blot analysis of extracts from various cell lines, serum-starved overnight (-) followed by treatment with Human Interferon-α1 #8927 (hIFN-α1, 10 ng/ml, 15 min; +) or Human Interleukin-4 #8919 (hIL-4, 10 ng/ml, 10 min; +) using Phospho-Jak1 (Tyr1034/1035) (D7N4Z) Rabbit mAb (upper)

Jak1. Western blot analysis of JAK1 on Jurkat cell lysate.

pSTAT6. Western blot analysis of extracts from ACHN cells, untreated (-) or treated with Human Interleukin-4 (hIL-4) #8919 (10 ng/ml, 10 min; +), and BaF3 cells, untreated (-) or treated with Mouse Interleukin-3 (mIL-3) #8923 (10 ng/ml, 5 min; +), using Phospho-Stat6 (Tyr641) (D8S9Y) Rabbit mAb (upper), Stat (D3H4) Rabbit mAb #5397 (middle), and β-Actin (D6A8) Rabbit mAb #8457 (lower).

STAT6. Western blot analysis of extracts from various cell lines using Stat6 (D3H4) Rabbit mAb.

pIL-4Ra. Western blot analysis of Phospho-IL-4 Receptor/CD124 pTyr497 in extracts from 293 cells using a Phospho-IL-4 Receptor/CD124 pTyr497 polyclonal antibody (Product # PA5-38614).

Beta-Actin. Western blot analysis of β-Actin expression in HeLa (A), Jurkat (B), K-562 (C) and A-431 (D) whole cell lysates.

In addition most of flow antibodies described above were used in previous studies published from the lab (Dar et al. PLos Biol. 2021, Layman et al. Nat Comm 2017)providing further validation to the antibodies.

For western blot experiments we have detected proteins at appropriate molecular weight which was consistent with the molecular weight shown in vendor(s) website and its predicted molecular weight based on the amino acid sequence. In addition we have used antibody from more than one vendor to confirm the specificity and in most cases both antibodies showed band at the same position. Cul5 and CIS antibodies were further validated by Immunoprecipitation followed by mass-spectrometry. CIS antibody from SantaCruz Biotechnology showed only one band around 32 kDa while CIS antibody from Cell Signaling Technology showed two bands around 32 and 37 Kda.

Eukaryotic cell lines

Policy information about cell lines

Cell line source(s)	D10 from ATCC
Authentication	Cell line was authenticated by ATCC by performing STR profiling
Mycoplasma contamination	No Mycoplasma contamination was detected by ATCC
Commonly misidentified lines (See ICLAC register)	No misidentified cell line was used

Animals and other organisms

Policy information about studies involving animals; ARRIVE guidelines recommended for reporting animal research

Laboratory animals	C57BL/6, CD45.1, CD45.2 and Rag1 KO mice were used in the experiments. Mice from both genders were used for experiments. Mice were either 8-10 week old or 30-36 old weeks old. 6-10 week old Rag KO mice were used as recipient for bone marrow chimera experiments. All mice were housed at a temperature of 72 °F and humidity of 30–70% in a 12-h light/dark cycle with ad libitum access to food and water.

Wild animals	No wild animals were used in this study
Field-collected samples	No field collected samples were used in this study
Ethics oversight	All procedures in mice were performed in accordance to protocols approved by the Children hospital of Philadelphia IACUC. All animal experiments were performed according to the protocols approved by the Institutional Animal Care and Use Commitee of NIAID, NIH

Note that full information on the approval of the study protocol must also be provided in the manuscript.

Flow Cytometry

Plots

Confirm that:

- [x] The axis labels state the marker and fluorochrome used (e.g. CD4-FITC).
- [x] The axis scales are clearly visible. Include numbers along axes only for bottom left plot of group (a 'group' is an analysis of identical markers).
- [x] All plots are contour plots with outliers or pseudocolor plots.
- [x] A numerical value for number of cells or percentage (with statistics) is provided.

Methodology

Sample preparation	Spleen, lymph nodes and lungs were harvested from mice after euthanasia by carbon dioxide and cervical dislocation. Lungs were treated with collagenase I and Ia (Sigma, catalogue number C0130 and C9891 respectively), and 20ug/ml DNAse I (Roche, catalogue number 10104159001) in 10ml plain RPMI for 1hr at room temperature prior to homogenization. Homogenates were treated with 2ml of RBC lysis buffer for 2min and passed through an additional 70µm filter. For intracellular staining, 5*106 cells from these homogenates were resuspended in 1ml of RPMI 1640 supplemented with 10% FCS, 100U/ml Penicillin-streptomycin, 1X Glutamax (ThermoFisher, cat. 35050061), 1X Non-essential amino acids (Gibco, cat. 11140-050), 2mM HEPES (Gibco, cat. 15630-080), 1mM sodium pyruvate (Corning, cat. 20115013), 8l/L 2-mercaptoethanol (Sigma), 1g/ml Golgi Plug (BD, cat. 555029), 1g/ml Golgi Stop (BD, cat. 554724), PMA (30ng/ml, Calbiochem) and Ionomycin (1µM, Abcam). These resuspensions were incubated at 37°C for 4hrs before proceeding with staining.<br><br>Single cell suspensions were first stained with a fixable viability dye (Life Technologies, cat. L34961) and pre-treated with unlabeled anti-CD16/CD32 (Fc Block, BD Pharminogen, cat. 564219). Cells were surface stained for 15 mins at 4oC in FACS buffer with the following antibodies: anti-CD4 (BioLegend, clone GK1.5), anti-CD3 (BioLegend or BD biosciences, clone 17A2), anti-CD62L (BioLegend, clone MEL-14), anti-CD44 (BioLegend, clone IM7), anti-CD45.1 (BioLegend, clone A20), and anti-CD45.2 (BioLegend, clone 104), anti-CD124 (BD, mIL4R-M1). After staining cells were then washed with FACS buffer and fixed in BD Cytofix/Cytoperm (BD, cat 554714) for 20 mins. For intracellular cytokine staining cells were washed in BD perm wah buffer and stained for intracellular cytokine in BD perm wash buffer (cat 554723) with following antibodies: anti-IFN-y (BD, XMG1.2), anti-IL2 (BL, JES6-5H4), anti-IL5 (BD, TRFK5), anti-IL4 (BD, 11B11), anti-IL13 (ebioscience; eBio13A), anti-IL17A (BL, TC11-18H10.1), antiIL-9 (BL, RM9A4). For mast cell staining lung homogenate was stained with anti-CD45 (Biolegend, 30 F-11), anti-FceRI (ebiosciences, Mar-1) and anti-CD117 (Biolegend, 2-B8) for 15 mins at 4oC and fixed using BD cytofix/ cytoperm buffer. For eosinophils lung homogenate was stained with anti-CD45 (Biolegend, 30 F-11), anti-Siglec F (BD, E50-2440), anti-CD11c (Biolegend, N418) and anti-CD11b (Biolegend, M1/70) for 15min at 4oC and fixed using BD cytofix/ cytoperm buffer.
Instrument	For acquisition BD LSR Fortessa instruments were used.
Software	Flow data was analysed using FlowJo Software
Cell population abundance	Cells were not sorted for the experiments provided in this manuscript.
Gating strategy	For Flow analysis cells were gated first on Lymphocyte population using FSC/SSC after which cells were gates on Singlets using SSC-A/SSC-H and FSC-A/FSC-H. After signlets live cells were gates based on live-Dead stain using Live-Dead Blue dye. Live-Dead negative cells were gated on CD3 to identity lymphocytes after which they were gated on CD4/CD8. CD4 cells were subsequently gated on CD4/CD44 to identify activated CD4 T cells and then cells were gated in CD44/Cytokines (IL-4, IL-5, IL-9, IL-17, IFN-y).

- [x] Tick this box to confirm that a figure exemplifying the gating strategy is provided in the Supplementary Information.
